# Evaluation of Computer‐aided Detection for Identifying Missed Gastric Cancer After Endoscopic Submucosal Dissection

**DOI:** 10.1002/deo2.70357

**Published:** 2026-08-17

**Authors:** Tatsunori Minamide, Tomonori Sato, Seiichiro Sakaguchi, Noboru Kawata, Yuki Maeda, Masao Yoshida, Yoichi Yamamoto, Taishi Okumura, Tetsuya Suwa, Hiroshi Ashizawa, Kohei Shigeta, Haruka Nakamura, Akifumi Notsu, Toshio Uraoka, Hiroyuki Ono

**Affiliations:** ^1^ Division of Endoscopy Shizuoka Cancer Center Shizuoka Japan; ^2^ Department of Gastroenterology IMSUT Hospital The Institute of Medical Science The University of Tokyo Tokyo Japan; ^3^ Advanced Technology Research Division Olympus Corporation Tokyo Japan; ^4^ Clinical Research Center Shizuoka Cancer Center Shizuoka Japan; ^5^ Department of Gastroenterology and Hepatology Gunma University Graduate School of Medicine Maebashi Japan

**Keywords:** artificial intelligence, endoscopy, gastric cancer, missed diagnosis, surveillance

## Abstract

**Objectives:**

Computer‐aided detection (CADe) using deep learning is promising for reducing missed gastric cancers (MGCs) and supporting physicians in double‐checking endoscopic images. We aimed to evaluate the CADe efficacy for MGCs after endoscopic submucosal dissection (ESD).

**Methods:**

We collected 2324 endoscopic images, including 60 of MGCs detected during surveillance esophagogastroduodenoscopy within 18 months after initial ESD. The performance of the CADe system, developed for early GC detection using deep learning, was compared with that of 10 endoscopists in a detection study using collected images. Per‐lesion sensitivity, per‐image detection performance, and diagnostic time were compared.

**Results:**

The per‐lesion sensitivity for MGCs was 15.0% (9/60) and 13.8% (83/600) for CADe and endoscopist detection, respectively (*p* = 0.538). The respective per‐image computer‐aided and endoscopist performance sensitivity was 11.3% and 10.6% (*p* = 0.548), specificity 88.5% and 94.8% (*p* < 0.001), positive predictive value 4.4% and 8.3%, and negative predictive value 95.7% and 96.0%. The per‐image CADe time was significantly shorter (0.03 s vs. 2.86 s, *p* < 0.001). CADe showed higher sensitivity in certain subgroups, although these findings should be interpreted cautiously given the small sample size.

**Conclusions:**

No significant difference in sensitivity was observed between CADe and endoscopist detection for MGCs after ESD, and the absolute sensitivity remained low. Further improvements are needed before clinical implementation of CADe as a double‐checking tool.

**Trial Registration:**

The authors have confirmed clinical trial registration is not needed for this submission.

## 1 Introduction

Gastric cancer (GC) is the fifth leading cause of cancer‐related death worldwide, with approximately one million new cases diagnosed annually [1]. Early detection considerably improves prognosis, with a 5‐year survival rate of nearly 90% in early‐stage disease [2]. Esophagogastroduodenoscopy (EGD) is widely used for screening early GC (EGC) and reduces mortality [3, 4, 5, 6]. When EGC is detected, minimally invasive endoscopic treatments such as endoscopic submucosal dissection (ESD) can be performed instead of surgery [7].

Even after curative ESD for EGC, regular endoscopic surveillance is crucial because of the high risk of metachronous lesions, with a 5‐year cumulative incidence of 9.5%–20.8% [8, 9, 10, 11]. However, surveillance is limited by missed GC (MGC), occurring in 3.7%–9.0% of cases after ESD [8, 11, 12]. To reduce MGCs, all surveillance EGD images can be double‐checked, as in Japanese screening programs; however, this is impractical because of the workload on physicians.

Computer‐aided detection (CADe) systems using deep learning have shown promise in supporting endoscopists in detecting GC [13, 14, 15, 16]. These systems can automatically detect and annotate lesions in still images, videos, and real‐time endoscopy. CADe may reduce MGCs and enhance daily practice as a double‐checking tool; however, few studies have evaluated its performance in MGCs. Therefore, we aimed to assess the efficacy of CADe compared with that of endoscopists in detecting MGCs after ESD.

## 2 Methods

### 2.1 Study Population

This retrospective study compared lesion detection performances of CADe and endoscopists for MGCs using endoscopic images from patients who underwent ESD for EGC in a naïve stomach (no prior gastric resection) at the Shizuoka Cancer Center between January 2009 and June 2018. MGCs were defined as new EGCs detected during surveillance EGD within 18 months after initial ESD. We excluded patients who underwent additional gastrectomy, those with MGCs not retrospectively identifiable on pre‐ESD EGD images, those lacking accurate biopsy confirmation before ESD, inadequate EGD preparation before ESD, and those with local recurrence after ESD.

**FIGURE 1:**
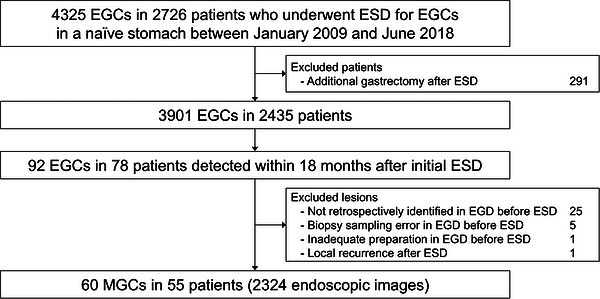
Patient flowchart. EGC, early gastric cancer; ESD, endoscopic submucosal dissection; MGC, missed gastric cancer.

ESD for EGCs in a naïve stomach was performed in 4325 lesions in 2726 patients. After excluding 424 EGCs in 291 patients who underwent gastrectomy, 3901 lesions in 2435 patients remained. Among these, 92 EGCs in 78 patients were identified within 18 months after initial ESD. After applying exclusion criteria, 60 MGCs in 55 patients were included (Figure 1).

### 2.2 Endoscopic Images and Clinicopathological Data of MGCs

We collected 2324 white‐light images of the entire stomach obtained immediately before ESD during EGD in 55 cases with 60 MGCs, excluding 230 images containing the index EGCs. EGDs were performed using a systematic screening protocol for the stomach (SSS) [17]. All images were obtained using GIF‐H290Z (EVIS LUCERA ELITE) and GIF‐H260Z (EVIS LUCERA; Olympus Corporation, Tokyo, Japan). Clinicopathological data included tumor location, macroscopic type, *Helicobacter pylori* status, histopathology, invasion depth, tumor size, and ulceration, collected from electronic records and classified according to the Japanese classification of gastric carcinoma [18]. *H. pylori* status was defined as uninfected (negative serology, urea breath test, or stool antigen and no eradication history), infected (any positive test), or eradicated (confirmed successful eradication by urea breath test or stool antigen) [19].

### 2.3 CADe Development

The CADe system (Olympus Corporation) was developed for real‐time EGC detection using deep learning with supervised learning and YOLO v3 (yolov3‐voc) [20], which enables faster and more accurate object detection than conventional methods. The target was defined as gastric lesions with macroscopic features suggestive of EGC. Training data included endoscopic videos of 760 target lesions and 591 non‐lesion cases collected outside this study. Board‐certified endoscopists annotated consecutive frames and delineated lesion boundaries.

CADe performance was evaluated using 95 additional EGC videos (20 fps) as a validation dataset. The system marked candidate lesions as bounding rectangles. As previously reported, successful detection was defined as a rectangle overlapping the reference annotation for at least three consecutive frames [21]. Using this criterion, per‐lesion sensitivity for target lesions was 93.7% (95% confidence interval [CI], 86.8–97.7%).

### 2.4 Detection Study of MGCs

Following CADe development, a detection study of MGCs using CADe and endoscopists was conducted on endoscopic images collected between June and August 2024. Ten endoscopists participated, including five trainees and five experts with <10 and ≥10 years of experience in GC endoscopic diagnosis, respectively. GC presence was assessed on each endoscopic image that partially contained MGCs. All clinicopathological data were blinded, and only white‐light images were used. Images were maintained at original resolution (96 dpi) and resized to 416 × 416 pixels for CADe input. Detected lesions were annotated with bounding rectangles, and intersection over union (IoU) with reference annotations was calculated. The time required to review all endoscopic images was measured.

Referenced annotations were based on histopathological findings after ESD and prepared by a board‐certified expert endoscopist (Tatsunori Minamide), not involved in the detection study. Olympus Corporation employees (Tomonori Sato and Seiichiro Sakaguchi) did not participate in annotation and had no access to clinicopathological data during the process.

### 2.5 Endpoints

The primary endpoint was per‐lesion sensitivity for MGCs, defined as correctly detected lesions divided by MGCs. Successful detection was defined as IoU >0.2 on at least one image of the lesion. This relatively lenient threshold was selected to reflect the study objective of evaluating CADe as an initial cue for lesion recognition rather than precise delineation. In MGCs, partial overlap may be sufficient to attract an endoscopist's attention and prompt further inspection. Secondary endpoints included per‐image detection performance (sensitivity, specificity, positive predictive value [PPV], and negative predictive value [NPV]) and per‐image diagnostic time. W Subgroup analyses were performed according to clinicopathological characteristics among CADe, trainees, and experts.

### 2.6 Statistical Analyses

Categorical variables are expressed as frequencies (%) and continuous variables as medians (interquartile ranges) or means (standard deviations [SDs]). Detection performance was reported with 95% CIs. The McNemar test was used for sensitivity and specificity comparisons, and the Student's *t*‐test was used for per‐image diagnostic time. A *P* value <0.05 was considered statistically significant. Analyses were performed using EZR version 1.68 (Saitama Medical Center, Jichi Medical University, Saitama, Japan).

For sensitivity and specificity comparisons, paired data were created by matching CADe output with each endoscopist's assessment for the same image; thus, each image contributed multiple paired observations corresponding to each endoscopist. The McNemar test was applied to these paired datasets.

### 2.7 Ethics

This study was approved by the institutional review board of Shizuoka Cancer Center (registration number: 2022‐29‐2022‐1‐3, approval date: December 9, 2022) and conducted in accordance with the Declaration of Helsinki. Informed consent was obtained using an opt‐out method.

## 3 Results

### 3.1 Clinicopathological Characteristics of MGCs

Table 1 summarizes the clinicopathological characteristics of 60 MGCs. Approximately half of the lesions were located in the middle third of the stomach (46.7%), most frequently along the lesser curvature (31.7%). The predominant macroscopic type was 0‐IIc (63.3%). *H. pylori* infection has been eradicated in 28.3% of cases. Most lesions were differentiated‐type (91.7%), pT1a (95.0%), and ulceration‐negative (96.7%), with a median tumor size of 12.5 mm.

**TABLE 1:** Clinicopathological characteristics of missed gastric cancers (MGCs).

Characteristics	*n* = 60
Tumor location 1	
Upper third	13 (21.7)
Middle third	28 (46.7)
Lower third	19 (31.7)
Tumor location 2	
Lesser curvature	19 (31.7)
Anterior wall	13 (21.7)
Greater curvature	13 (21.7)
Posterior wall	15 (25.0)
Macroscopic type	
0‐IIa	21 (35.0)
0‐IIb	1 (1.7)
0‐IIc	38 (63.3)
*Helicobacter pylori* infection status	
Uninfected	3 (5.0)
Infected	6 (10.0)
Eradicated	17 (28.3)
Unknown	34 (56.7)
Histopathological type	
Differentiated	55 (91.7)
Undifferentiated	5 (8.3)
Invasion depth	
pT1a (M)	57 (95.0)
pT1b1 (SM1)	1 (1.7)
pT1b2 (SM2)	2 (3.3)
Tumor size, mm	12.5 (7.0–18.0)
Ulceration	
Positive	2 (3.3)
Negative	58 (96.7)

*Note*: Values are presented as n (%) or medians (interquartile ranges).

Abbreviations: M, mucosa; MGC, missed gastric cancer; SM, submucosa.

### 3.2 Detection Performance for MGCs by CADe and Endoscopists

Table 2 shows detection performance for MGC by CADe and endoscopists. No significant difference in per‐lesion sensitivity was observed (15.0% [9/60] vs. 13.8% [83/600], *p* = 0.538). Similarly, per‐image sensitivity did not differ significantly (11.3% vs. 10.6%, *p* = 0.548). Specificity was significantly higher for endoscopists than for CADe (88.5% vs. 94.8%, *p* < 0.001). PPV was higher for endoscopists (4.4% vs. 8.3%), although a statistical comparison was not performed. NPV was comparable between groups (95.7% vs. 96.0%). Per‐image diagnostic time was significantly shorter for CADe than for endoscopists (0.03 ± 0.00 s vs. 2.86 ± 1.21 s, *p* < 0.001). The mean IoU of correctly detected MGCs was 0.61 ± 0.18 for CADe and 0.51 ± 0.18 for endoscopists.

**TABLE 2:** Detection performance for missed gastric cancers (MGCs) by computer‐aided detection (CADe) and endoscopists.

	CADe	Endoscopists	*p*‐value
Per‐lesion analysis			
Sensitivity	15.0 (7.1–26.6) 9/60	13.8 (11.2–16.9) 83/600	0.538
Per‐image analysis			
Sensitivity	11.3 (6.0–18.9) 12/106	10.6 (8.8–12.6) 112/1060	0.548
Specificity	88.5 (87.1–89.8) 1964/2218	94.8 (94.5–95.1) 21023/22180	< 0.001
PPV	4.4 (2.3–7.6) 12/272	8.3 (6.9–10.0) 112/1342	N/A
NPV	95.7 (94.7–96.5) 1964/2052	96.0 (95.7–96.3) 21023/21898	N/A
Diagnostic time, s	0.03 (0.00)	2.86 (1.21)	< 0.001

*Note*: Values are expressed as percentages (95% CI), n/n, or means (standard deviations).

Abbreviations: CI, confidence interval; CADe, computer‐aided detection; MGC, missed gastric cancer; N/A, not applicable; NPV, negative predictive value; PPV, positive predictive value.

### 3.3 Per‐lesion Sensitivity for MGCs Based on Clinicopathological Characteristics

Table 3 shows per‐lesion sensitivity according to clinicopathological characteristics. CADe exhibited higher sensitivity for lesions in the upper third (23.1% vs. 11.5%, *p* = 0.001) and along the lesser curvature (26.3% vs. 15.8%, *p* = 0.003), whereas endoscopists showed higher sensitivity for lesions in the middle third (10.7% vs. 16.8%, *P* = 0.020). CADe also performed better in *H. pylori*‐uninfected cases (66.7% vs. 40.0%, *p* = 0.013). Histopathologically, CADe revealed higher sensitivity for pT1b lesions (66.7% vs. 30.0%, *p* = 0.003).

**TABLE 3:** Per‐lesion sensitivity for missed gastric cancers (MGCs) based on clinicopathological characteristics.

	CADe	Endoscopists	*p*‐Value
Tumor location 1			
Upper third	23.1 (3/13)	11.5 (15/130)	0.001
Middle third	10.7 (3/28)	16.8 (47/280)	0.020
Lower third	15.8 (3/19)	11.1 (21/190)	0.137
Tumor location 2			
Lesser curvature	26.3 (5/19)	15.8 (30/190)	0.003
Anterior wall	7.7 (1/13)	6.9 (9/130)	1.000
Greater curvature	15.4 (2/13)	23.1 (30/130)	0.100
Posterior wall	6.7 (1/15)	9.3 (14/150)	0.453
Macroscopic type			
0‐IIa	9.5 (2/21)	12.9 (27/210)	0.248
0‐IIb	0 (0/1)	0 (0/10)	N/A
0‐IIc	18.4 (7/38)	14.7 (56/380)	0.115
*Helicobacter pylori* infection status			
Uninfected	66.7 (2/3)	40.0 (12/30)	0.013
Infected	0 (0/6)	11.7 (7/60)	N/A
Eradicated	5.9 (1/17)	10.0 (17/170)	0.190
Histopathological type			
Differentiated	16.4 (9/55)	14.0 (77/550)	0.203
Undifferentiated	0 (0/5)	12.0 (6/50)	N/A
Invasion depth			
pT1a (M)	12.3 (7/57)	13.0 (74/570)	0.743
pT1b (SM)	66.7 (2/3)	30.0 (9/30)	0.003
Tumor size			
0–9 mm	14.3 (3/21)	16.7 (35/210)	0.568
10–19 mm	7.7 (2/26)	6.9 (18/260)	0.814
≥ 20 mm	30.8 (4/13)	23.0 (30/130)	0.089
Ulceration			
Negative	13.8 (8/58)	13.1 (76/580)	0.754
Positive	50.0 (1/2)	35.0 (7/20)	0.248

*Note*: Values are presented as % *(n/n)*.

Abbreviations: CADe, computer‐aided detection; M, mucosa; MGC, missed gastric cancer; N/A, not applicable; SM, submucosa.

### 3.4 Detection Performance Among CADe, Trainees, and Experts

Table 4 compares detection performance among CADe, trainees, and experts. No significant differences were observed in per‐lesion sensitivity (CADe 15.0%, trainees 15.0%, experts 12.7%). Per‐image sensitivity showed no significant differences (CADe 11.3%, trainees 10.9%, experts 10.2%). Specificity was significantly higher for trainees (93.7%) and experts (95.9%) than for CADe (88.5%). PPV and NPV were higher for experts and trainees than for CADe. Per‐image diagnostic time was significantly shorter for CADe (0.03 s) than for trainees (3.41 s) and experts (2.31 s).

**TABLE 4:** Detection performance for missed gastric cancers (MGCs) compared among computer‐aided detection (CADe), trainees, and experts.

	CADe	Trainees	Experts	*p*‐value (CADe vs. trainees)	*p*‐value (CADe vs. experts)	*p*‐value (trainees vs. experts)
Per‐lesion analysis						
Sensitivity	15.0 (9/60)	15.0 (45/300)	12.7 (38/300)	1.000	0.360	0.427
Per‐image analysis						
Sensitivity	11.3 (12/106)	10.9 (58/530)	10.2 (54/530)	0.907	0.525	0.749
Specificity	88.5 (1964/2218)	93.7 (10393/11090)	95.9 (10630/11090)	< 0.001	< 0.001	< 0.001
PPV	4.4 (12/272)	7.3 (58/797)	9.9 (54/545)	N/A	N/A	N/A
NPV	95.7 (1964/2052)	96.0 (10393/10823)	96.0 (10630/11075)	N/A	N/A	N/A
Diagnostic time, s	0.03 (0.00)	3.41 (1.34)	2.31 (0.88)	< 0.001	< 0.001	0.163

*Note*: Values are presented as % (n/n) or means (standard deviations).

Abbreviations: CADe, computer‐aided detection; MGC, missed gastric cancer; N/A, not applicable; NPV, negative predictive value; PPV, positive predictive value.

### 3.5 Causes of False Positives by CADe

Table 5 shows that the most common cause of false positives was gastritis‐related changes (redness, atrophy, intestinal metaplasia), accounting for 58.3% of cases. Other causes included normal anatomical structures (cardia, pylorus, and angulus; 6.7%), hyperplastic polyps (5.9%), and scarring (5.1%).

**TABLE 5:** Causes of false positives by computer‐aided detection (CADe).

Causes of false positives	*n* = 254
Gastritis (redness, atrophy, and intestinal metaplasia)	148 (58.3)
Normal anatomical structure (cardia, pylorus, and angulus)	17 (6.7)
Hyperplastic polyp	15 (5.9)
Scar	13 (5.1)
Blood	10 (3.9)
Fold	9 (3.5)
Submucosal tumor	7 (2.8)
Xanthoma	7 (2.8)
Mucus	6 (2.4)
Ulcer	6 (2.4)
Extrinsic compression	5 (2.0)
Halation	5 (2.0)
Foam	3 (1.2)
Vessel	2 (0.8)
Suction mark	1 (0.4)

*Note*: Values are presented as *n* (%).

Abbreviation: CADe, computer‐aided detection.

### 3.6 Representative Cases

**FIGURE 2:**
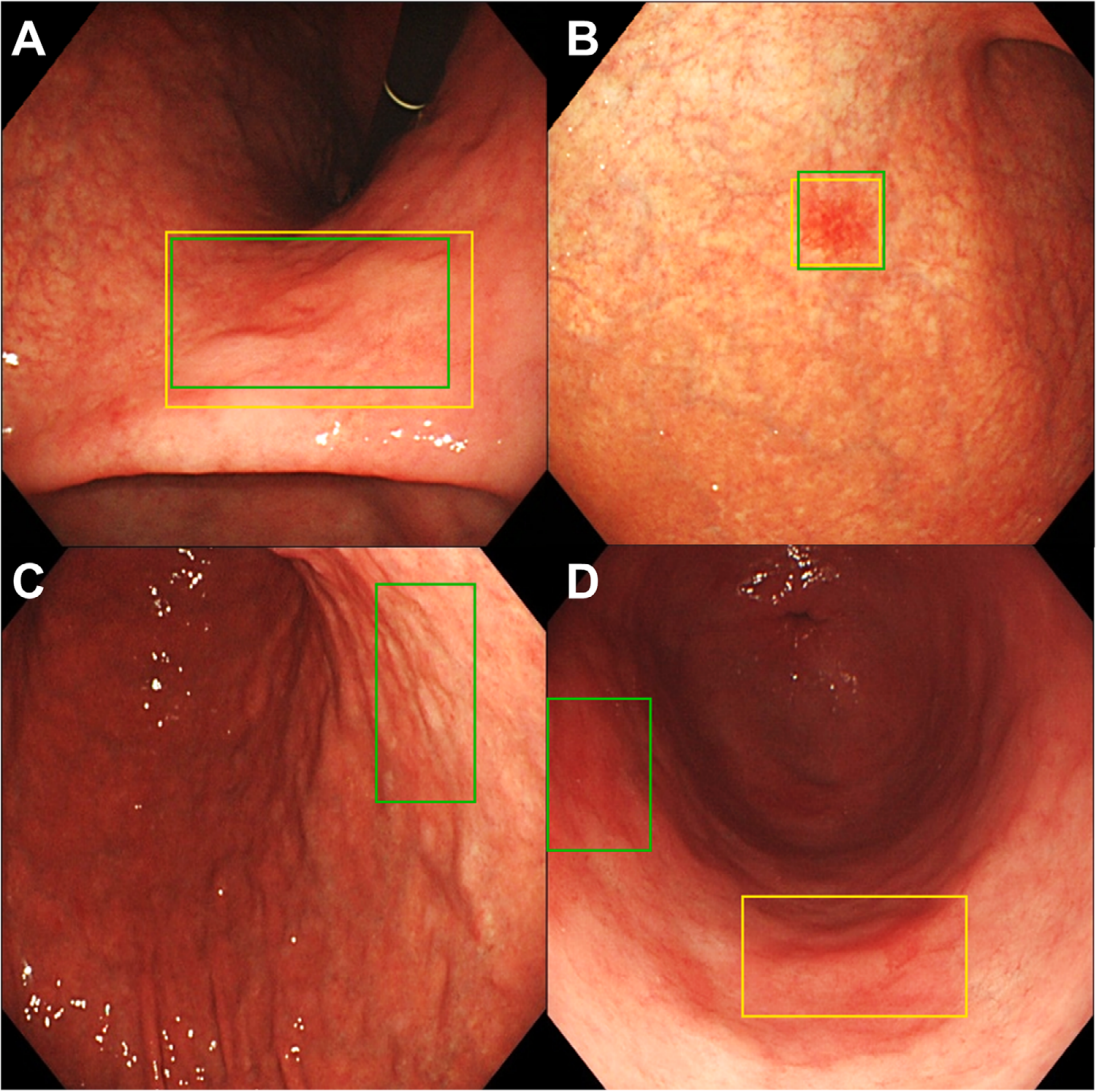
Representative cases of successful and unsuccessful detection of MGC by CADe. (a) and (b) show endoscopic images of successful detection of MGC by CADe, whereas (c) and (d) show unsuccessful detection. (a) A 15‐mm 0‐IIa lesion in the lesser curvature of the middle third, with minimal color change or irregularity. (b) A 10‐mm erythematous 0‐IIc lesion in the anterior wall of the middle third that must be differentiated from inflammation. (c) A 20‐mm 0‐IIc lesion in the posterior wall of the middle third. However, the CADe failed to detect this. (d) A 12‐mm 0‐IIc lesion on the anterior wall of the lower third. The CADe incorrectly detected an erythematous depressed lesion in the greater curvature. The green boxes represent the correct annotations, and the yellow boxes represent the annotations determined by CADe. CADe, computer‐aided detection; MGC, missed gastric cancer.

Figure 2 presents representative cases of successful and unsuccessful MGC detection using CADe. Successful cases include a 15‐mm 0‐IIa lesion in the middle third lesser curvature showing subtle color change and irregularity, and a 10‐mm erythematous 0‐IIc lesion in the middle third anterior wall requiring differentiation from inflammation. Histopathology confirmed a differentiated pT1a lesion in the latter.

Unsuccessful cases include a 20‐mm 0‐IIc lesion in the middle third posterior wall that was not detected by CADe, and a 12‐mm 0‐IIc lesion in the middle third anterior wall that was incorrectly annotated as an erythematous depressed lesion in the greater curvature. Both were confirmed as differentiated pT1a lesions after ESD.

## 4 Discussion

We evaluated the efficacy of CADe in detecting MGCs after ESD, a particularly challenging setting for lesion recognition. No significant difference in per‐lesion sensitivity was observed between CADe and endoscopists, and overall sensitivity remained low. Although CADe significantly reduced diagnostic time compared with endoscopists, its specificity was significantly lower, resulting in more false positives. Therefore, further improvements are needed before CADe can be used as a double‐checking tool for endoscopic images in clinical practice.

Recent studies from Japan and China have reported high CADe performance for EGCs detection, with per‐lesion sensitivities of 92.2% for still images and 87.8% for videos [13, 15]. A randomized controlled trial further demonstrated that CADe significantly reduced gastric neoplasm miss rates (risk ratio 0.224) [16]. In contrast, the per‐lesion sensitivity for MGCs in this study was only 15.0%, slightly higher than that of endoscopists (13.8%). Unlike prior studies focusing on typical EGCs, we evaluated MGCs, which are more subtle and frequently overlooked lesions. Therefore, our findings highlight the limitations of current CADe systems in real‐world clinical scenarios. The low sensitivity observed in both CADe and endoscopists underscores the inherent difficulty of detecting these lesions. Future development should incorporate more subtle and easily missed lesions into training datasets to improve robustness. To our knowledge, this is the first study assessing CADe performance specifically for MGCs after ESD.

Regarding CADe as a double‐checking tool, Oura et al. [22] reported excellent performance for lesion detection and identification of low‐quality images, with per‐image sensitivity of 93.0% (1602/1723) and per‐lesion sensitivity of 84.0% (42/50), supporting its potential utility. However, our study focused specifically on post‐ESD MGCs identified after expert endoscopic review, providing a more stringent assessment of CADe performance in missed lesions. Despite these differences, both studies demonstrated substantially shorter diagnostic times with CADe than with endoscopists, highlighting its potential to support double‐checking. In this study, CADe processed images approximately 100 times faster than endoscopists (0.03 s vs. 2.86 s per image). Therefore, CADe holds promise as a double‐checking tool due to its rapid processing capability and independence from fatigue, even when handling large volumes of endoscopic images.

Aside from per‐lesion sensitivity and diagnostic time, CADe showed significantly lower per‐image specificity than endoscopists (88.5% vs. 94.8%, *p* < 0.001), and the absolute PPV was also low. This raises concerns about an increased false‐positive rate when used for double‐checking. Although higher sensitivity is crucial for reducing MGCs, improving specificity is equally important for minimizing MGCs, improving specificity is equally important to avoid unnecessary physician workload and biopsies in clinical practice. As previously reported, the most common causes of CADe false positives are gastritis, followed by normal anatomical structures [13, 23]. Therefore, training CADe on a large and diverse dataset of non‐cancerous images, including various forms of gastritis and normal anatomy, is necessary to reduce false positives.

Our group previously reported four endoscopic causes of MGCs after ESD: perceptual error, exposure error, sampling error, and inadequate preparation [12]. Among these, perceptual error was the most common (69%), with risk factors including male sex, isochromatic lesions, location in the greater curvature, and tumor size <12 mm. Here, we excluded MGC attributable to exposure error (not retrospectively identifiable on pre‐EGD ESD), sampling error, and inadequate preparation to focus on perceptual errors, which are potentially preventable by CADe. Accordingly, 25 cases due to exposure error, five due to sampling error, and one due to inadequate preparation were excluded. The final analysis included 60 MGCs due to perceptual errors. Given these etiologies, CADe alone may be insufficient to reduce MGCs in surveillance EGDs, and careful inspection, appropriate sampling, and adequate preparation remain essential.

In the clinicopathological analysis, CADe revealed significantly higher sensitivity for lesions in the upper third of the stomach. As MGCs are frequently located in this region [8, 24], CADe may help reduce perceptual errors in this anatomically challenging area. In contrast, endoscopists showed higher sensitivity in the middle third, suggesting that a combined strategy may improve overall detection performance. CADe may therefore be particularly useful as a double‐check tool in difficult‐to‐visualize regions such as the upper stomach. Notably, CADe showed higher sensitivity in *H. pylori*–uninfected cases, possibly because the absence of background inflammation and mucosal heterogeneity facilitates the detection of subtle abnormalities. In contrast, such lesions may be less familiar to endoscopists and more easily overlooked. Although the number of cases was limited, it is clinically relevant that CADe identified more pT1b cancers than endoscopists, as these lesions may progress to advanced disease before the next EGD [25].

No significant difference was observed in per‐lesion sensitivity for MGCs among CADe, trainees, and experts. Kato et al. [8] reported that limited experience (<500 EGD cases) is an independent risk factor for oversight, whereas Raftopoulos et al. [26] found no association with experience level. Here, endoscopists with <10 years of GC diagnostic experience were classified as trainees. However, their overall EGD experience was likely higher than in previous studies, which may have minimized differences between groups. Nevertheless, although not significant, experts tended to require less diagnostic time than trainees, suggesting that experience may still influence MGC diagnosis.

Our study has some limitations. First, it was a single‐center study using the SSS method for image acquisition [17]. which is not universally adopted, limiting the generalizability. Second, sample size calculations were not possible due to the absence of prior data on MGC detection, resulting in a small sample size and potentially limited statistical power. Multicenter studies with larger datasets are needed for validation. Third, MGCs were defined as EGCs detected within 18 months after ESD. However, definitions vary (12–36 months in previous studies) [8, 11, 12, 26, 27]. Although this reflects institutional practice, it may include both missed synchronous lesions and early metachronous cancers, introducing heterogeneity that could affect performance. Fourth, although CADe was developed using video data, this study used still images only, limiting external validity compared with real‐time or video‐based endoscopy. Lack of temporal and multi‐angle information may have underestimated performance. However, this reflects clinical practice where still images are often used for double‐checking, suggesting CADe may be more suitable for post‐procedural review than for real‐time use. Fifth, McNemar's test was applied to paired CADe–endoscopist data; however, this approach does not fully account for clustering within images and readers, and may underestimate variance. More robust methods, such as generalized estimating equations, would be preferable. Sixth, the CADe system was based on YOLO v3, an earlier architecture, and the low sensitivity may partly reflect model limitations rather than intrinsic CADe performance for subtle lesions such as MGCs. Finally, several subgroup analyses included very small numbers (e.g., *H. pylori*–uninfected cases and pT1b lesions). These results should therefore be interpreted cautiously as exploratory and require validation in larger studies.

In conclusion, CADe did not outperform endoscopists in detecting MGCs after ESD and showed limited sensitivity. Although CADe markedly reduced diagnostic time, further algorithm refinement is required before serving as a reliable double‐checking tool in real‐world clinical practice.

## Author Contributions

Tatsunori Minamide and Hiroyuki Ono conceived and designed the study. Noboru Kawata, Yuki Maeda, Masao Yoshida, Yoichi Yamamoto, Taishi Okumura, Tetsuya Suwa, Hiroshi Ashizawa, Kohei Shigeta, Haruka Nakamura, and Hiroyuki Ono participated in the detection study. Tomonori Sato and Seiichiro Sakaguchi conducted the detection study using computer‐aided detection and calculated the data. Tatsunori Minamide, Tomonori Sato, Seiichiro Sakaguchi, Noboru Kawata, Yuki Maeda, Masao Yoshida, Yoichi Yamamoto, Akifumi Notsu, Toshio Uraoka, and Hiroyuki Ono performed data analysis and interpretation. Akifumi Notsu supported the statistical analyses. Tatsunori Minamide drafted the manuscript. Tomonori Sato, Seiichiro Sakaguchi, Noboru Kawata, Yuki Maeda, Masao Yoshida, Yoichi Yamamoto, Taishi Okumura, Tetsuya Suwa, Hiroshi Ashizawa, Kohei Shigeta, Haruka Nakamura, Akifumi Notsu, Toshio Uraoka, and Hiroyuki Ono critically revised the article for important intellectual content. All the authors have read and approved the final version of this manuscript.

## Funding

The authors have nothing to report.

## Ethics Statement

**Approval of the research protocol by an Institutional Reviewer Board**: The Institutional Review Board of Shizuoka Cancer Center approved this study (approval number: 2022‐29‐2022‐1‐3, approval date: December 9, 2022).

## Consent

Informed consent was obtained through opt‐out methods.

## Conflicts of Interest

Tomonori Sato and Seiichiro Sakaguchi provided the computer‐aided detection system developed by Olympus Corporation free of charge under a joint research agreement. Their roles were limited to technical support for the CADe system and data processing. They were not involved in establishing the reference standard or outcome assessment. Toshio Uraoka received a speaker honorarium from Olympus Corporation. The other authors declare no conflicts of interest.

## Data Availability

The data that support the findings of this study are not publicly available due to privacy reasons, but are available from the corresponding author upon request.
